# Publication Characteristics of Foot and Ankle Trauma Publications: A Review of Articles From 1997 to 2017

**DOI:** 10.7759/cureus.12607

**Published:** 2021-01-10

**Authors:** Nicholas A Andrews, Bradley Alexander, James Jones, Abhinav Agarwal, Achraf H Jardaly, Gerald McGwin, Ashish Shah

**Affiliations:** 1 Orthopaedic Surgery, University of Alabama at Birmingham, Birmingham, USA; 2 Epidemiology, University of Alabama at Birmingham, Birmingham, USA

**Keywords:** foot and ankle surgery, trauma surgery, publication trends, orthopedic surgery

## Abstract

Background

The purpose of this study is to evaluate and compare publishing characteristics in foot and ankle trauma articles published in two subspecialty journals and two general orthopedic journals.

Methods

All trauma articles related to foot and ankle surgery published from five different time intervals over a 20-year period were collected and the following was analyzed: authorship, level of evidence, type of study, citations, and geographic region.

Results

Foot and Ankle International (FAI) had the highest percentage of last and corresponding authors that were fellowship-trained in foot and ankle. The Journal of Bone and Joint Surgery American and British volumes (JBJS(A) and JBJS(B), respectively) and the Journal of Orthopaedic Trauma (JOT) articles had a higher percentage of last and corresponding authors that were fellowship-trained in trauma.

Conclusion

Foot and ankle-trained authors are currently under-represented in foot and ankle trauma literature. As the field of foot and ankle continues to grow, it is important that the experts in the field are well represented in the literature.

## Introduction

As the field of foot and ankle surgery continues to grow and progress, it is essential that the quality of research in this area does the same. With more academic literature being published each year [1], greater emphasis is continually being put on the quality of clinical research and supporting evidence in published articles. Several studies in orthopedic literature have noted the increase in number of authors over time, level of evidence (LOE), and number of international author groups [2-8]. To date, there are no studies evaluating publications covering traumatic foot and ankle conditions and how these publications vary amongst different journals. 

The purpose of this study is to evaluate and compare publishing characteristics in foot and ankle trauma articles published in two subspecialty journals, Foot and Ankle International (FAI) and the Journal of Orthopaedic Trauma (JOT), and two general orthopedic journals, the Journal of Bone and Joint Surgery American and British volumes (JBJS(A) and JBJS(B), respectively), at five different time intervals over a 20-year period. Publishing characteristics analyzed included authorship, level of evidence, article type, citations, and geographic origin of authors trends. 

This review will provide insight into the quality of foot and ankle trauma literature being published in a variety of orthopedic journals and see if similar characteristics exist between journals.

## Materials and methods

Article collection

Using the FAI, JOT, JBJS(A), and JBJS(B) archives, we collected all trauma articles related to foot and ankle surgery articles published from five different time intervals over a 20-year period. Journal selection was decided by impact factor. JOT and FAI represent the highest impact factors for trauma and foot and ankle publications, respectively. JBJS(A) holds the highest impact factor for general orthopedic journals [9]. Due to the low amount of trauma-related foot and ankle articles published in JBJS(A), JBJS(B) was also included to increase the amount of reviewed articles from a general orthopedic journal. The intervals included: January 1, 1997 to December 31, 1997; January 1, 2002 to December 31, 2002; January 1, 2007 to December 31, 2007; January 1, 2012 to December 31, 2012; and January 1, 2017 to December 31, 2017. 

Exclusions and citations

Inclusion criteria included all trauma articles related to foot and ankle surgery from the years 1997, 2002, 2007, 2012, and 2017. Editorials, letters to the editor, announcements, technical notes, events, errata, retracted manuscripts, historical papers, and articles covering chronic foot pathology were excluded. After exclusions were made there were 147 foot and ankle articles published in FAI, JOT, JBJS(A), and JBJS(B) over the 20-year period. Information related to each article including number of authors, the highest level of education of the authors that were publishing, study design, and country of origin was recorded. Additionally, a Google Scholar search was conducted for each article to determine the number of times an article had been cited [10]. 

Fellowship and attending status

The American Board of Orthopaedics Surgery certificate verification tool was used to determine if an author was a resident or attending. To account for the lag time between residency and board certification we counted authors as attendings only if they had been certified for greater than two years at the date of article publication. To determine the fellowship training of authors, all authors that completed their residency in the United States were then searched in Google® using their name combined with: “orthopedic trauma fellowship,” “orthopedic foot and ankle fellowship,” “orthopedic fellowship,” the name of the institution that was associated with their published article, and “fellowship.” The first two pages of search results were viewed. Only articles from the United States were analyzed for author fellowship training due to variations in training globally. 

Review of level of evidence

Two reviewers independently reviewed the articles and assigned an LOE and article type. The level of evidence was assigned on a scale of I to V. Level I was the highest grade that an article could receive. For the articles that received two different LOE grades, a discussion was had between the two reviewers until a decision was reached. If the two reviewers could not reach an agreement, they had a discussion with the senior author, and the methodology proposed by Spindler et al. was used to make a final decision [11]. Small case series of five or fewer patients that were a compilation of multiple case reports were assigned as “case reports,” despite having more than two patients. These studies were assigned an LOE grade of V. Larger case series reporting on patient cohorts were labeled as either a therapeutic, prognostic, or diagnostic study. These studies were assigned an LOE grade of IV. Animal, cadaver, basic science articles, and case reports were included and were assigned an LOE of V.

Statistical analysis

The kappa coefficient was used to measure interobserver agreement for LOE. Publication characteristics were compared over time and with respect to LOE. All categorical variables were compared using chi-square and Fisher’s exact tests (when individual cell count was less than five); the Kruskal-Wallis test was used for comparisons involving count variables.

## Results

Interobserver agreement

The kappa value for the interobserver reliability of the LOE for each article, k = 0.96, showed an almost perfect agreement between the reviewers.

Authorship

The median number of authors was highest at five in JBJS(A)(B) despite not hitting significance (Table 1). FAI had a significantly higher median (0) and mean (0.708) number of MD/PhDs (p= .031 and .018, respectively), but no other authorship trends differed significantly between the four journals. JBJS(A)(B) had the highest percentage of last and corresponding authors that were PhDs (16.7% and 12.5%, respectively). 

**Table 1:** Author credentials by journal FAI: Foot and Ankle International; JOT: Journal of Orthopaedic Trauma (JOT); JBJS(A) and JBJS(B): Journal of Bone and Joint Surgery American and British volumes

	JOT	FAI	JBJS (A)(B)	P value
Median Number of Total Authors	4	4	5	0.328
Education Characteristics				
Highest Degree of Last Author	N (%)	N (%)	N (%)	
MD (or equivalent)	41 (82.0)	50 (78.1)	16 (66.7)	0.0874
MD/PhD	4 (8.0)	1 (1.6)	0	
PhD	3 (6.0)	4 (6.3)	4 (16.7)	
Other	2 (4.0)	9 (14.1)	4 (16.7)	
Highest Degree of Corresponding Author	N (%)	N (%)	N (%)	
MD (or equivalent)	46 (90.2)	55 (90.2)	18 (75.0)	0.0708
MD (or equivalent)/PhD	0	2 (3.3)	0	
PhD	2 (3.9)	0	3 (12.5)	
Other	3 (5.9)	4 (6.6)	3 (12.5)	
Median no of MDs	4	3	3	0.257
Median no of MD/PhDs	0	0	0	0.031
Mean no of MD/PhDs	0.180	0.708	0.203	0.018
Median no of PhDs	0	0	0	0.691
Median no of DPMs	0	0	0	0.686
Median no of all other Degrees	0	0	0.5	0.098

Fellowship 

The results for fellowship training only pertain to articles that were connected to institutions within the United States. Eighty-one articles were analyzed (Table 2). JBJS(A)(B) articles had a higher percentage of last (72.7%) and corresponding authors (45.5%) that were fellowship-trained in trauma (p= .009 and p= .028, respectively). FAI had the highest percentage of last (28.6%) and corresponding authors (36.1%) that were fellowship-trained in foot and ankle (p= .011 and .0003, respectively). The median number of authors that were residents was one for each journal. The median number of authors that were attendings was highest for JOT at three. 

**Table 2:** Fellowship information and level of training FAI: Foot and Ankle International; JOT: Journal of Orthopaedic Trauma (JOT); JBJS(A) and JBJS(B): Journal of Bone and Joint Surgery American and British volumes

	JOT	FAI	JBJS (A)(B)	P value
Fellowship Last Author (Articles from United States)	21 (%)	21 (%)	10 (%)	
Trauma	16 (50.0)	8 (22.9)	8 (72.7)	0.009
Foot & Ankle	2 (6.3)	10 (28.6)	0 (0)	0.011
Both	3 (9.4)	3 (8.6)	2 (18.2)	0.690
Fellowship Corresponding Author (Articles from United States)	19 (%)	22 (%)	5 (%)	
Trauma	16 (50.0)	6 (17.1)	5 (45.5)	0.028
Foot & Ankle	1 (3.1)	13 (36.1)	0 (0)	0.0003
Both	2 (6.3)	3 (8.6)	0 (0)	0.582
	JOT	FAI	JBJS (A)(B)	P value
United States Articles only	N	N	N	
Median no of Residents	1	1	1	0.351
Median no of Attendings	3	2	2	0.108

Level of evidence and type of study

The percentage of level I (12.5%) and II (12.5%) articles were highest in JBJS(A)(B) (Table 3). FAI and JBJS(A)(B) had a higher percentage of level III evidence articles (25.0% and 29.17%, respectively) compared to JOT (13.56%). The distribution of level V evidence articles was greater in JOT (37.29%) compared to FAI and JBJS(A)(B) (23.44% and 0%, respectively). 

The four journals, FAI, JOT, and JBJS(A)(B), had a similar distribution of clinical therapeutic and clinical diagnostic studies (Table 3). JBJS(A)(B) had a greater number of clinical prognostic studies (50.0%) compared to JOT and FAI (8.47% and 21.88%, respectively). There were no case reports in JBJS(A)(B). The percentage of anatomic studies was highest in JOT at 8.47%. 

**Table 3:** Study characteristics by journal FAI: Foot and Ankle International; JOT: Journal of Orthopaedic Trauma (JOT); JBJS(A) and JBJS(B): Journal of Bone and Joint Surgery American and British volumes

Level of Evidence by Reviewer	JOT N (%)	FAI N (%)	JBJS(A)(B) N (%)	P value
I	6 (10.17)	4 (6.25)	3 (12.50)	
II	3 (5.08)	5 (7.81)	3 (12.50)	
III	8 (13.56)	16 (25.00)	7 (29.17)	0.0547
IV	20 (33.90)	24 (37.50)	11 (45.83)	
V	22 (37.29)	15 (23.44)	0 (0)	
Type of Study				
Clinical Therapeutic	23 (38.98)	28 (43.75)	10 (41.67)	
Clinical Diagnostic	7 (11.86)	6 (9.38)	2 (8.33)	
Clinical Prognostic	5 (8.47)	14 (21.88)	12 (50.00)	0.0003
Case Report	8 (13.56)	12 (18.75)	0 (0)	
Anatomic Study (Biomechanical)	5 (8.47)	1 (1.56)	0 (0)	
Reviews and Other	11 (18.64)	3 (4.69)	0 (0)	
Median Citations Per year	3.71	2.96	5.33	0.022
Geographical Region				
United States	32 (54.2)	35 (54.7)	11 (45.8)	
Europe	13 (22.0)	20 (31.3)	10 (41.7)	
Asia	3 (5.1)	6 (9.4)	2 (8.3)	0.1881
Other	7 (11.9)	2 (3.1)	1 (4.2)	
Intercontinental collaboration	4 (6.8)	1 (1.6)	0 (0)	

Citations and geographic region

The median number of citations per year per article was significantly higher in JBJS(A)(B) (p= .022) (Table 3). A majority of articles published in each journal were from groups based in the United States. JBJS(A)(B) had the highest percent of articles from European countries compared to FAI and JOT (Table 3). FAI had the highest percent of articles from Asian countries (9.4%), and JOT had the highest percent of articles that were the result of international collaboration (6.8%).

Publication trends over time

Several trends were noted amongst all articles across five time intervals over a 20-year period (Table 4). A noticeable shift in LOE of the articles being published occurred, as a smaller percentage of articles being published recently feature Level 1 evidence compared to 20 years ago. Level I evidence articles decreased from 14.81% of articles in 1997 to 4.88% in 2017. Instead, more level III and IV evidence articles are being published. In 2017, level III and IV evidence articles comprised 43.9% and 41.46% of all the articles in this study, respectively. There was a significant decrease in the amount of level V evidence articles being published from 48.15% in 1997 to 9.76% in 2017 (p= .0006). 

The number of case reports has decreased from 33.33% of published articles in 1997 to 2.44% in 2017 (Table 4). Anatomic and biomechanical studies have also seen a decrease from 11.11% in 1997 to 2.44% in 2017. As of 2017, a majority of clinical studies being published are therapeutic and prognostic. 

**Table 4:** Level of evidence and study type over time

	1997	2002	2007	2012	2017	P value
Level of Evidence by Reviewer	N (%)	N (%)	N (%)	N (%)	N (%)	
I	4 (14.81)	2 (9.52)	2 (6.90)	3 (10.34)	2 (4.88)	
II	3 (7.41)	4 (19.05)	1 (3.45)	4 (13.79)	0 (0)	
III	1 (3.70)	2 (9.52)	3 (10.34)	7 (24.14)	18 (43.90)	0.0006
IV	7 (25.93)	8 (38.10)	13 (44.83)	10 (34.46)	17 (41.46)	
V	13 (48.15)	5 (23.81)	10 (34.46)	5 (17.24)	4 (9.76)	
Type of Study	N (%)	N (%)	N (%)	N (%)	N (%)	
Clinical Therapeutic	9 (33.33)	14 (66.67)	5 (17.24)	17 (58.62)	16 (39.02)	
Clinical Diagnostic	3 (11.11)	0 (0)	4 (13.79)	2 (6.90)	6 (14.63)	
Clinical Prognostic	2 (7.41)	2 (9.52)	9 (31.03)	4 (13.79)	14 (34.15)	0.0005
Case Report	9 (33.33)	4 (19.05)	5 (17.24)	1 (3.45)	1 (2.44)	
Anatomic Study (Biomechanical)	3 (11.11)	0 (0)	2 (6.90)	0 (0)	1 (2.44)	
Reviews and Other	1 (3.70)	1 (4.76)	4 (13.79)	5 (17.24)	3 (7.32)	

## Discussion

While the orthopedic subspecialty of foot and ankle continues to advance and grow, it is important that a high quality of research in this field is maintained; however, there are no studies to date evaluating the quality, primarily based on LOE and citation counts, of research in foot and ankle trauma publications. This study is novel in both this regard and in its analysis of fellowship training of authors. 

By evaluating the fellowship training of the last and corresponding author, it is possible to gain greater insight into the perspective from which many of the studies are being conducted. It can be reasonably presumed that orthopedic specialists, whether in foot and ankle or trauma, are more inclined to conduct research with principles and ideas that are related to the training they received during fellowship. This is compared to authors who did not receive fellowship training after completion of their residency.

The goal of this study is to compare two subspecialty journals, FAI and JOT, and two general orthopedic journals, JBJS(A)(B), in order to see any differences in publishing characteristics between the journals through evaluating authorship characteristics, LOE, type of study, and citations across the four journals and over a 20-year time period. Additionally, this study has identified temporal trends across all articles. This review provides a comprehensive view of the current state of foot and ankle trauma research and the direction that it is headed.

Authorship and fellowship training 

Between the journals, no major trends in the distribution of authors were noted, but while the median number of MD/PhD authors per article was zero for all four journals, the mean number of authors with an MD/PhD was significantly higher in JBJS(A)(B). The increased involvement of MD/PhDs may lead to higher-quality publications. These authors may draw on their PhD training to design larger prospective studies. Each journal exhibited similar numbers per article of MDs, PhDs, and other degrees. The similarities between journals are comparable to the authorship characteristics found in other orthopedic specialties and journals [2,12]. 

Comparing fellowship training data across journals, FAI shows a significantly greater proportion of articles by foot and ankle-trained authors than either JOT or JBJS(A)(B), but these proportions highlight the relatively low overall percentage of foot and ankle-trained last and corresponding authors (28.6% and 36.1%, respectively). Conversely, JOT and JBJS(A)(B) have significantly greater proportions of articles by trauma-trained authors than FAI. Fellowship training of authors was only analyzed for United States articles in this study, and the status of fellowship training of authors in non-United States articles is unknown. Of note, no other studies evaluating the fellowship status of authors in orthopedic literature were found to use as a comparison, so average proportions of fellowship-trained authors in the literature are largely unknown at the present time. The overall lack of articles by foot and ankle-trained authors compared to trauma specialists, however, shows the current under-representation of publications by these experts. The need for foot and ankle specialists to be better represented in foot and ankle trauma literature is evident and important for a more comprehensive perspective on this important topic. 

Level of evidence and study design

Consistent with previous orthopedic studies of similar design, the greatest proportion of publications in all four journals in the study were level III evidence and lower [3,4]. FAI and JOT both had high percentages of level V evidence studies at 23.4% and 37.3%, respectively. Various reasons have been proposed for the consistently high number of lower evidence articles including the difficulty of randomized controlled trials (RCTs) in surgical studies and the relative financial and time advantage of lower LOE studies compared to RCTs [4]. Due to the nature of trauma research, cadaver and biomechanical studies can provide valuable insight and surgical guidance. When considering the subject matter of JOT, the fairly high number of level V evidence studies is reasonable. Interestingly, a greater proportion of level I and II evidence articles was seen in each journal in this analysis than in a foot and ankle LOE study looking at the same journals [4]. 

It should be noted that a study being a lower level of evidence does not inherently mean that it is a lesser study. The value of high level of evidence studies should not be disregarded. These studies use less mechanism-based reasoning and are more clinically applicable; however, all study types have a role in the literature and help the field advance and grow. 

Type of study 

A majority of articles were clinical therapeutic in nature for each journal, making up at least 38% of the articles in all four journals. These results agree with the findings of similar trend studies in orthopedics that analyzed study type [3,4]. The emphasis on this study type demonstrates the importance of identifying and improving treatment methods within the field of orthopedics. There was a significant difference in the amount of clinical prognostic studies published by each journal. JBJS(A)(B) articles published the highest amount of prognostic studies (50%) which was significantly higher than both FAI and JOT. The prominent number of clinical prognostic studies in both FAI and JBJS(A)(B) highlight the academic nature of these journals in their commitment to publishing studies that provide long-term follow-up data to accurately reflect the course of orthopedic injuries. Over the last 20 years, it is clear that FAI and JBJS(A)(B) have made prognostic studies a priority. 

Only 8.5% of JOT articles were clinical prognostic, but fewer prognostic studies are expected in a trauma journal due to the acute nature of traumatic injuries as a whole. Overall, it is promising that greater than half of the articles for each journal were clinical in nature and directly focused on improving the care of patients through clinical evidence rather than mechanism-based evidence. 

Citations and geographic region 

JBJS(A)(B) displayed a significantly higher median number of citations/year than FAI and JOT. These results are not unique to this paper and have been seen in other studies [4]. The higher number of citations per year for JBJS(A)(B) may be attributed to a combination of several factors. It is a well-respected and recognized orthopedic journal that publishes articles covering a broad range of topics within orthopedic surgery. Additionally, JBJS(A)(B) had an increased distribution of high LOE studies (level I and II) compared to the two subspecialty journals. Large prospective studies with higher LOE grades are often well known throughout the field and may be more commonly cited than low LOE studies like case reports and expert opinions. Nevertheless, an equal distribution of each journal was seen among the top ten most cited articles in the study. The number of times an article is cited is not a direct indicator of quality but can be used to estimate the significance and prevalence of the topic in the field. Foot and ankle trauma article citation rates were found to be equivalent or superior to rates in other orthopedic journals and subspecialties [10,13,14].

North American countries published the highest number of articles in each journal; however, all four journals showed a sizeable amount of publications from various countries throughout the world. JBJS(A)(B) had the highest percent of articles from European countries compared to FAI and JOT, and FAI had the highest percent of articles from Asian countries (9.4%). These results are consistent with a prior study looking at global publication trends from 1995 to 2015 that showed the United States as the largest producer of publications with global publishing gradually increasing [8]. The increasing globalization of authorship speaks to the international readership and influence of the journals in this study.

Publication trends over time

In this study, a significant increase in level III and IV studies was observed from 1997 to 2017. A decrease was also seen in level V evidence studies. However, there was a decrease in the percentage of high-level studies (level I and II) over the 20-year period as well. Compared to other LOE trend studies in orthopedics, the results of this study are unique and show trends unlike several other studies that found increases in high LOE studies or decreases in level III and IV studies [3,4,6]. The trends of this study suggest a possible shift in foot and ankle trauma research from studies that draw on mechanism-based reasoning to form conclusions to large, retrospective cohort studies and case series. An example of this shift is the companion journal created by JBJS(A) for case reports, JBJS Case Connector, established in 2011. The lack of high-level evidence studies could be due to the difficultly in recruiting patients prospectively that have undergone a traumatic orthopedic emergency. There is still room for improvement with regards to high-level clinical trials in foot and ankle trauma publications, but based on the findings of this study and others in varying areas of orthopedics, it is encouraging to see that the publishing trends are going in a positive direction and emphasizing a clinical focus [2,6].

Strengths and limitations

The purpose of this study is to compare foot and ankle trauma articles between four prominent journals and analyze trends over a 20-year period of time. Although many studies of a similar nature have involved analyzing temporal trends or journal comparison, this study is able to accomplish both in a single study. Key trends and characteristics in foot and ankle trauma articles over a 20-year period were identified and will help contribute to the literature in addition to helping understand the current state of foot and ankle trauma research. 

While the primary reviewers diligently searched the article archives of each journal, it is possible that suitable articles could have been missed or removed from the list of articles during the article collection process. Additionally, it was not feasible to review all foot and ankle trauma articles across multiple journals. Although each of the four journals included in the study has a high impact factor within the field of orthopedics, articles from other well-respected and notable journals that would have been a good representation of their respective field were not analyzed. 

One limitation of the study is that in sampling articles in one out of every five-year interval without analyzing the intermediate years, it is possible that the data does not exactly match the trends or comparisons made during the study period. Fellowship status was classified only based on the last author and the corresponding author of each article; other coauthors that have received fellowship training may have been involved in study design. However, the training of the last or corresponding author is a suitable approximation of the vantage point of the article being published. Lastly, fellowship training was also only analyzed for articles published in the United States. 

## Conclusions

FAI and JOT are two of the leading subspecialty journals for foot and ankle and trauma publications, respectively. JBJS(A) and JBJS(B) are representative of leading general orthopedic journals, and many individuals use these journals to guide clinical practice. This study demonstrates that these journals have comparable publishing characteristics and that both the foot and ankle and trauma subspecialty journals are publishing articles with similar characteristics of those published in JBJS(A)(B). 

Although these journals exhibit fairly similar publishing characteristics, it is essential that foot and ankle literature continues to progress in order to better guide clinical practice and decision making. The increase in articles assigned a LOE grade of III and IV found in this study suggest a shift in foot and ankle trauma research toward a more clinical focus; however, for the field to continue to grow the emphasis on high-quality publications needs to continue. Foot and ankle fellowship-trained authors are currently under-represented in foot and ankle trauma literature. It is important that foot and ankle specialists are well represented in the literature for a more complete understanding of the topic.
